# Books and Authors

**Published:** 1908-11

**Authors:** 


### BOOKS AND AUTHORS.

---

**Pulmonary Tuberculosis and its Complications.** By **Sherman G. Bonney,** M.D., Professor of Medicine, Denver and Gross College of Medicine, Denver. Octavo of 778 pages, with 189 original illustrations, including 20 in colors and 60 x-ray photographs. Philadelphia and London: W. B. Saunders Company, 1908. (Cloth, $7.00; half morocco, $8.50 net prices.)

At no time in the history of medicine has such interest in pulmonary tuberculosis, both in professional and lay circles been manifest as at present. The public in general is beginning to understand that it is a preventable disease and the inquiry is making on all sides "how shall preventable measures best be adopted to reach the largest number of persons especially those with slender purses?" The recent congress at Washington will have missed its purpose if it has not answered this question, at least in large measure.

The work, the treatise perhaps we should say, now submitted for the consideration of the profession, is essentially a clinical exposition of the subject, intended more particularly for the general practitioner and from this viewpoint possesses special value. The residence of the author within the zone of climatic prevention or cure, qualifies him to speak with intelligence on this part of the subject,—a part, too, of no mean import. Since the isolation of the tubercle bacillus, views relating to the cause of pulmonary consumption, have completely changed front, the doctrine of heredity even by many having been turned away as unworthy of credence.

These questions are thoroughly discussed by Bonney, he holding to the conservative viewpoint—namely, that congenital tuberculosis must be an admitted possibility, though of exceedingly rare occurrence. All avenues of infection are considered, means of early diagnosis set forth, and methods of cure are described with great exactitude, all however, with simplicity. It is a book that keeps abreast of scientific facts and is an ornament to the literature of medicine, to which it contributes a fund of information. Every physician who wishes to keep himself thoroughly informed regarding all phases of tuberculosis of the pulmonary variety, should give careful study to the book; it is full of interest and the author in the last chapter gives his observations concerning the practical application of vaccine therapy to this disease.

**State Board Questions and Answers.** By **R. Max Goepp,** M.D., professor of clinical medicine at the Philadelphia Polyclinic. Octavo, pp. 684. Philadelphia and London: W. B. Saunders Co. (Cloth, $4.00.)

This book displays a surprising energy on the part of the compiler and is well worthy the attention of medical examiners —of anybody whose duties require them to examine for degrees or state license, or for the army or navy.

The topics covered are: physics, chemistry, physiology, anatomy, hygiene, materia medica and therapeutics, practice of medicine, surgery, obstetrics, gynecology, pathology, and bacteriology; and these are subdivided, as, for instance: practice of medicine into the main heading and general diagnosis, physical diagnosis, diseases of the respiratory organs; diseases of the heart and bloodvessels; the infectious diseases; the acute eruptive fevers; diseases of the digestive organs; diseases of the kidneys and bladder; the anemias; constitutional and nervous diseases. In short nothing of importance has been omitted. A valuable feature of the book is the full and complete index which makes it a work of easy reference.

---

**The Practical Medicine Series.** Ten volumes. Issued under the general editorial charge of **Gustavus P. Head,** M.D., Professor of Laryngology and Rhinology in the Chicago Post-Graduate Medical School. Vols. 1, and 2, General Medicine, and Surgery. Series 1908. Chicago: The Year Book Publishers. (Price, $1.50, $2.00; entire series, $10.00.)

1. General Medicine. This volume is edited by Frank Billings and J. H. Salisbury. It deals with diseases of the respiratory organs, circulatory organs, blood and bloodmaking organs, general infectious diseases, ductless glands, metabolic diseases, and diseases of the kidneys. Price, $1.50.

2. General Surgery. This number is edited by John B. Murphy, and is a compilation of the world's surgery during the past year (1907). The editor advocates a uniform technic in the administration of anesthetics. The entire work shows great skill in abstracting and is of great value for ready reference. Price, $2.00.

